# Racial and ethnic disparities in T helper 2-mediated inflammatory skin disorders

**DOI:** 10.1093/skinhd/vzag124

**Published:** 2026-07-31

**Authors:** Bracha L Pollack, Arielle N Roberts, Benjamin D Wagner, Gopika Ashokan, Xizhao Chen, Geoffrey E Hespe, Jinyeon Shin, Annica R Stull-Lane, Sarit Pal, Babak J Mehrara, Raghu P Kataru

**Affiliations:** Plastic and Reconstructive Surgery Service, Department of Surgery, Memorial Sloan Kettering Cancer Center, New York, NY, USA; Plastic and Reconstructive Surgery Service, Department of Surgery, Memorial Sloan Kettering Cancer Center, New York, NY, USA; Plastic and Reconstructive Surgery Service, Department of Surgery, Memorial Sloan Kettering Cancer Center, New York, NY, USA; Plastic and Reconstructive Surgery Service, Department of Surgery, Memorial Sloan Kettering Cancer Center, New York, NY, USA; Plastic and Reconstructive Surgery Service, Department of Surgery, Memorial Sloan Kettering Cancer Center, New York, NY, USA; Plastic and Reconstructive Surgery Service, Department of Surgery, Memorial Sloan Kettering Cancer Center, New York, NY, USA; Plastic and Reconstructive Surgery Service, Department of Surgery, Memorial Sloan Kettering Cancer Center, New York, NY, USA; Plastic and Reconstructive Surgery Service, Department of Surgery, Memorial Sloan Kettering Cancer Center, New York, NY, USA; Plastic and Reconstructive Surgery Service, Department of Surgery, Memorial Sloan Kettering Cancer Center, New York, NY, USA; Plastic and Reconstructive Surgery Service, Department of Surgery, Memorial Sloan Kettering Cancer Center, New York, NY, USA; Plastic and Reconstructive Surgery Service, Department of Surgery, Memorial Sloan Kettering Cancer Center, New York, NY, USA

## Abstract

T helper type 2 (Th2)-mediated inflammatory skin diseases, including atopic dermatitis, discoid lupus erythematosus, systemic sclerosis, autoimmune bullous diseases and secondary lymphoedema, demonstrate significant racial and ethnic disparities in prevalence, severity and clinical outcomes. These disorders share pathogenic mechanisms involving Th2-driven immune activation, fibrosis and epidermal barrier dysfunction, leading to chronic inflammation and substantial morbidity. Black and Hispanic populations consistently experience greater disease burden, more severe clinical manifestations and reduced quality of life compared with White patients, reflecting complex interactions between biologic and socioeconomic factors. Current molecular and immunological studies suggest that ancestry-associated differences in immune polarization, cytokine signalling, autoantibody expression, fibrotic pathways and epidermal barrier biology may contribute to variation in disease phenotype and severity across Th2-mediated disorders. Black patients have demonstrated enhanced Th2 and Th22-mediated inflammation, increased IgE production, elevated interleukin (IL)-4, IL-13 and IL-22 signalling, and distinct genetic and serological profiles, including variation in filaggrin-related barrier proteins and autoantibody expression. However, mechanistic studies directly evaluating race-associated Th2 immune responses remain limited in many inflammatory skin diseases, and under-representation of diverse populations in translational research and clinical trials continues to restrict understanding of disease biology and therapeutic response. This review synthesizes the current epidemiological, molecular and immunological evidence linking race, immune dysregulation and disease heterogeneity across Th2-mediated skin disorders. Together, these findings support a growing framework in which ancestry-associated differences in immune regulation and social determinants jointly shape disparities in Th2-mediated inflammatory skin disease.

## Teaser

Racial and ethnic disparities in dermatology extend far beyond access to care – they are deeply rooted in biology, genetics and immune regulation. This review explores how T helper type 2 (Th2)-driven inflammation contributes to unequal disease patterns across atopic dermatitis, discoid lupus erythematosus, systemic sclerosis, autoimmune bullous diseases and secondary lymphoedema. By integrating recent findings in immunology, molecular genetics and epidemiology, we reveal how enhanced Th2 polarization, cytokine signalling and structural skin differences contribute to the increased prevalence and severity of Th2-mediated inflammatory skin disorders among Black and Hispanic populations. These biologic variations intersect with socioeconomic inequities, delayed diagnosis and under-representation in clinical research, compounding the burden of disease. Through a multidisciplinary lens, this review underscores that reducing disparities requires more than equal access – it demands an inclusive understanding of how immune mechanisms and social factors converge to shape dermatological outcomes. By identifying knowledge gaps and advocating for diverse study participation, this work highlights critical opportunities to advance precision dermatology and promote skin health equity across populations.

## Introduction

Chronic inflammatory skin diseases profoundly affect people’s quality of life (QoL), increase morbidity and strain healthcare systems. They arise from immune dysregulation and epidermal barrier dysfunction, causing persistent physical and psychological distress. Racial and ethnic minority groups, particularly Black and Hispanic populations, experience a higher prevalence, greater severity and disparities in diagnosis and treatment.^1^ Patients with more richly pigmented skin tones also exhibit differences in epidermal barrier function, immune polarization and T helper type 2 (Th2)-mediated inflammation (Figure 1).^2^ These biologic differences may underlie distinct phenotypes and outcomes. Elevated systemic inflammation, including higher C-reactive protein (CRP) levels, has been observed in Black and Hispanic individuals.^3,4^ Black patients with breast cancer exhibit increased expression of interleukin (IL)-6 and resistin, an adipocyte-derived cytokine,^5^ while Black and US-born Hispanic adults show elevated IL-10 levels, indicating a heightened inflammatory state.^6^ Such baseline immune differences may influence disease risk, severity and treatment response.

**Figure 1:**
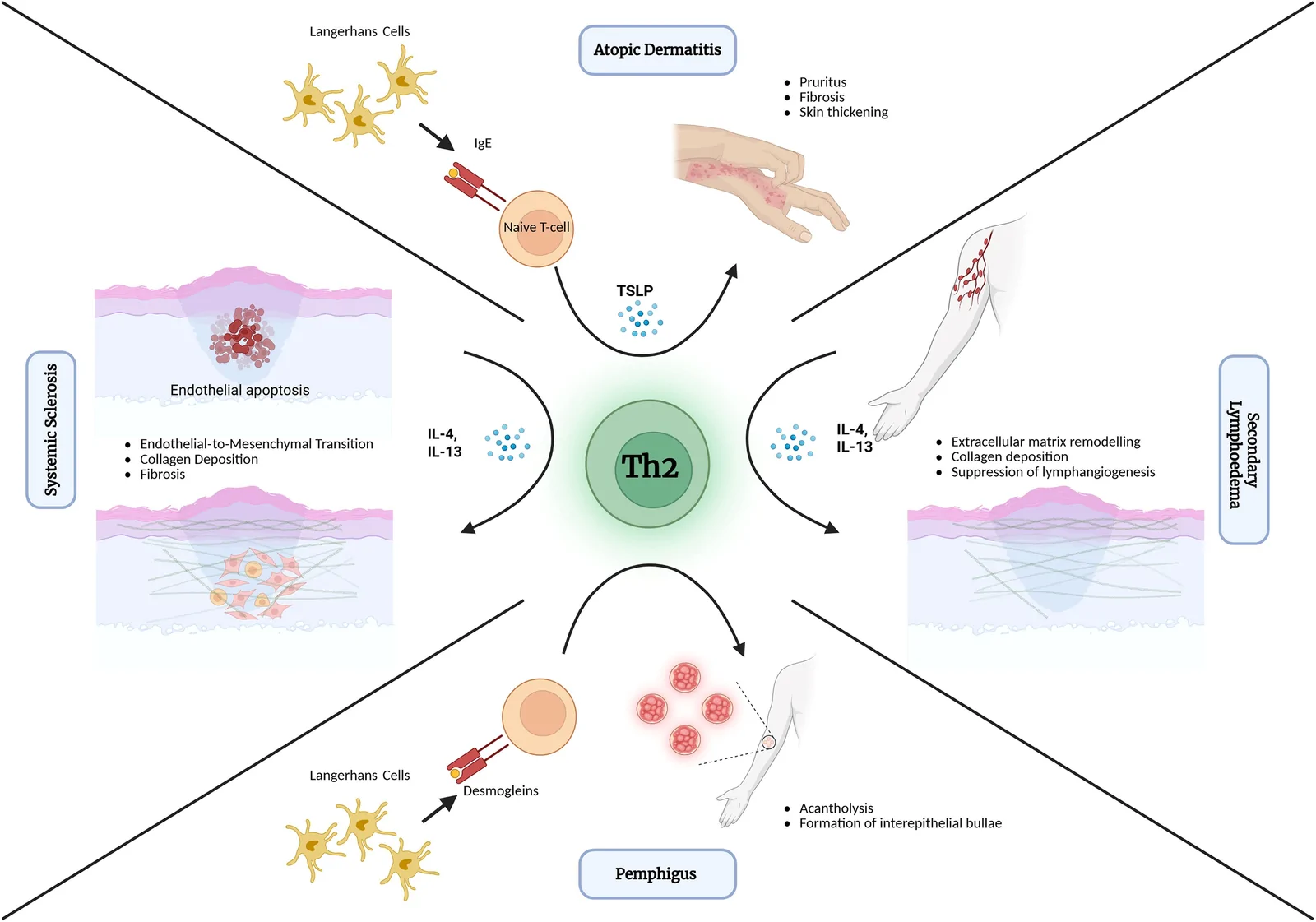
An overview of the role of T helper (Th)2 cells in the pathogenesis of various skin disorders. Th2 polarization and cytokine signalling underlie the pathogenesis of multiple inflammatory skin diseases, including atopic dermatitis (AD), systemic sclerosis (SSc), pemphigus and secondary lymphoedema. Th2 cells secrete interleukin (IL)-4 and IL-13, which collectively promote immune dysregulation, tissue remodelling and fibrosis. In AD, thymic stromal lymphopoietin (TSLP) released by keratinocytes drives Th2 activation, enhancing IgE-mediated sensitization, pruritus, fibrosis and epidermal thickening. In SSc, IL-4 and IL-13 induce endothelial apoptosis and endothelial-to-mesenchymal transition, leading to collagen deposition and dermal fibrosis. In secondary lymphoedema, the same cytokines promote extracellular matrix remodelling, excessive collagen accumulation and suppression of lymphangiogenesis, contributing to tissue fibrosis and swelling. In pemphigus, Langerhans cells present desmoglein autoantigens to naïve T cells, inducing Th2 polarization and autoantibody formation that result in acantholysis and intraepithelial blistering. Collectively, these interconnected Th2-driven mechanisms illustrate how overlapping cytokine pathways contribute to inflammation, fibrosis and barrier dysfunction across skin diseases in different ethnic groups. Illustrated with BioRender.

Although epidemiological disparities in inflammatory skin disease are well documented, comparatively fewer studies have directly examined race-associated differences in Th2 immune polarization, cytokine signalling and molecular endotypes. Existing evidence from atopic dermatitis (AD), systemic sclerosis (SSc) and autoimmune bullous diseases (AIBDs) suggests that ancestry-­associated variation in immune regulation, autoantibody expression, fibrotic signalling and epidermal barrier biology may ­contribute to heterogeneity in disease phenotype and severity. However, mechanistic studies specifically evaluating race-­stratified immune responses remain limited across many dermatological conditions. As such, this review synthesizes the available epidemiological, molecular and immunological data to explore how biologic heterogeneity and social determinants may collectively contribute to disparities in Th2-mediated skin disease.

## Methods

A narrative literature review was conducted to evaluate racial and ethnic disparities in Th2-mediated inflammatory skin disorders, with a focus on AD, discoid lupus erythematosus (DLE), SSc, AIBDs and secondary lymphoedema (SL). Relevant studies were identified using the PubMed/MEDLINE and Google Scholar databases for articles published in English from January 2005 to May 2025.

Disease-specific search terms included combinations of “racial disparities”, “ethnic disparities”, “Th2 inflammation”, “atopic dermatitis”, “discoid lupus erythematosus”, “systemic sclerosis”, “bullous pemphigoid”, “pemphigus”, “lymphedema”, “immune polarization”, “racial disparities”, “ethnic differences”, “Black”, “Hispanic”, “skin of color” and “cytokines”. Additional studies were identified through manual review of the reference lists in relevant articles.

Eligible articles included peer-reviewed human studies reporting epidemiology, pathophysiology, immune endotypes or clinical outcomes stratified by race or ethnicity, with preference given to more recent publications. Article selection was performed independently by two authors, with disagreements resolved through discussion and consensus with senior authors. Nonhuman studies, single case reports and conference abstracts without full manuscripts were excluded. The literature was synthesized narratively with an emphasis on Th2-mediated immune dysregulation, molecular heterogeneity and factors contributing to racial and ethnic differences in disease burden and outcomes.

## Atopic dermatitis

AD is one of the most extensively studied Th2-driven inflammatory skin disorders in the context of racial disparities. It is a chronic inflammatory condition that affects up to 10% of adults in the US and millions globally.^7,8^ AD is characterized by a relapsing–­remitting course of intense pruritus, excoriations and xerotic, leathery patches with papules.^9–11^ It can develop at any age and remains prevalent among adults.^12^ Moderate-to-severe AD presents therapeutic challenges, associated with increased rates of comorbidities, greater healthcare utilization and a heightened reliance on short-term disability benefits.^9^

### Pathophysiology

AD arises from complex interactions among epidermal barrier dysfunction, genetic susceptibility, environmental triggers and immune dysregulation. The disease is characterized predominately by Th2-mediated inflammation, with increased expression of IL-4, IL-5, IL-13 and IL-31 contributing to IgE production, pruritus, epidermal hyperplasia and chronic inflammation.^13–20^ Barrier dysfunction facilitates allergen sensitization and immune activation, while chronic disease demonstrates additional Th17, Th22 and Th1 pathway activation.^14,21–25^

### Racial disparities

Emerging evidence suggests that immune polarization and barrier abnormalities differ across racial and ethnic groups. *FLG* loss-of-function mutations, commonly associated with AD in European and Asian populations,^26–30^ are substantially less frequent among individuals of African descent.^29,31^ Instead, Black patients more commonly exhibit alterations in filaggrin-2 and claudin-1, which are associated with persistent and early-onset disease.^29,32^ Molecular profiling studies further demonstrate enhanced Th2 and Th22 polarization in Black patients, with elevated IL-22 expression contributing to epidermal thickening and impaired keratinocyte differentiation.^33–35^ Importantly, AD lesions in Black patients also demonstrate increased Th17 and selective Th1 activation, highlighting substantial immune heterogeneity and suggesting distinct inflammatory endotypes, compared with White patients.^36–38^ These molecular differences correlate with clinical phenotype. Black patients more commonly present with perifollicular papules, extensor involvement, xerosis and lichenification, whereas Asian patients frequently exhibit sharply demarcated and scaly lesions.^39–44^ Increased systemic inflammatory markers, including elevated erythrocyte sedimentation rate, CRP ferritin, eosinophils and serum IgE, have also been reported among African American patients, suggesting broader systemic immune activation that may contribute to increased disease severity and chronicity.^45^ Such immune heterogeneity may contribute not only to differences in clinical morphology and disease severity, but also to variable therapeutic response to targeted biologic therapies. However, racially stratified treatment response studies remain limited, demonstrating the need for more inclusive molecular and clinical research.

## Discoid lupus erythematosus

Systemic lupus erythematosus (SLE) is a multisystem autoimmune disease characterized by pathogenic autoantibody formation, immune complex deposition and Th2 skewing.^46,47^ Cutaneous lupus erythematosus may occur independently or in association with SLE, with DLE representing the most common chronic cutaneous subtype.^48^ Clinically, DLE presents with inflammatory plaques and papules that may progress to dyspigmentation, scarring and permanent alopecia, resulting in a substantial psychosocial and cosmetic burden.^49,50^

### Pathophysiology

DLE arises through complex interactions between genetic susceptibility, environmental triggers and immune dysregulation. Polymorphisms involving *TYK2*, *IRF5*, *CTLA4* and human leucocyte antigen (HLA) alleles have been associated with increased disease susceptibility,^51,52^ while ultraviolet radiation and smoking are recognized as environmental triggers.^53,54^ Immune-mediated keratinocyte injury promotes chronic inflammation characterized ­initially by Th2-skewed responses with progression towards Th1-mediated and cytotoxic inflammation in chronic disease.^54,55^ IL-21-producing Th cells, plasmacytoid dendritic cells, B-cell activation and tertiary lymphoid structure formation further contribute to sustained autoimmunity and tissue injury.^54,56^

### Racial disparities

DLE disproportionately affects Black and Hispanic populations. In Manhattan, New York, the prevalence of DLE has been reported to be highest in non-Latino Black (23.5) and Latino (8.2) individuals compared with White (1.8) and Asian (0.6) individuals.^57^ Meanwhile, studies in the southeastern USA have demonstrated a Black-to-White incidence ratio of 5.4.^48^ Black patients with SLE consistently demonstrate higher rates of DLE development and often present with more severe cutaneous disease.^57–61^

Importantly, disease phenotype differs across racial groups. Black patients are significantly more likely to develop scarring alopecia [odds ratio (OR) 3.00; *P* = 0.001], scalp dyspigmentation (OR 5.85; *P* < 0.001) and ear dyspigmentation (OR 2.89; *P* = 0.001),^62^ findings that may reflect an increased inflammatory burden and a greater clinical impact of pigment alteration in skin of colour. Diagnostic delays may also occur because erythema and active inflammation can be more difficult to recognize in people with darker skin tones, potentially contributing to progression towards irreversible scarring and dyspigmentation.

Despite marked epidemiological differences, mechanistic studies examining racial differences in DLE immune profiling and molecular endotypes remain limited. Existing evidence suggests that social determinants of health, including neighbourhood disadvantage and barriers to dermatological care, substantially contribute to disease severity and outcomes.^61^ However, the extent to which genetic susceptibility, cytokine polarization and immune heterogeneity contribute to racial differences in DLE progression remains poorly defined.

## Systemic sclerosis

SSc is a heterogeneous autoimmune disease characterized by systemic fibrosis, microvascular dysfunction and immune dysregulation affecting the skin and internal organs. Clinical manifestations range from limited cutaneous SSc to diffuse cutaneous SSc, the latter of which is associated with more extensive fibrosis and visceral involvement.^63–65^ Th2-mediated inflammation plays a central role in disease progression through fibroblast activation and extracellular matrix deposition.

### Pathophysiology

SSc pathogenesis involves complex interactions between endothelial injury, immune activation and progressive fibrosis. Early endothelial apoptosis promotes the recruitment of macrophages, B cells and T cells, leading to chronic inflammatory signalling and fibroblast activation.^65^ Th2 cytokines, including IL-4 and IL-13, drive collagen synthesis, extracellular matrix remodelling and the inhibition of collagen degradation, while IL-6, transforming growth factor-β and endothelin-1 further promote ­profibrotic responses.^65–68^ In addition to Th2 polarization, Th17 and T follicular helper cells contribute to immune heterogeneity and disease progression.^65,67,68^

### Racial disparities

Significant racial disparities exist in the prevalence, clinical phenotype and disease severity of SSc. Black patients develop SSc at a younger age and are more likely to present with diffuse cutaneous disease compared with White patients.^69,70^ They also experience more severe pulmonary fibrosis, skeletal muscle involvement and functional impairment, despite a shorter disease duration (1.33 vs. 0.86 years; *P* = 0.01).^70^ Higher modified Rodnan skin scores in Black patients further suggest increased fibrotic skin involvement and more aggressive disease phenotypes.^71,72^

Importantly, molecular and serological profiling studies suggest racial differences in immune endotypes. Black patients demonstrate higher frequencies of anti-topoisomerase I (26% vs. 17%), anti-U1 ribonucleic protein (RNP; 18% vs. 5%) and anti-U3 RNP (19% vs. 3%) compared with White patients (*P* < 0.001), ­profiles associated with more severe fibrosis, internal organ involvement and poorer clinical outcomes.^70^ Ethnicity-associated variation in HLA-linked autoantibody expression further supports underlying immunogenetic heterogeneity in SSc. In addition, several non-HLA susceptibility genes involved in immune regulation and fibrotic signalling, including *STAT4*, *IRF5*, *BANK1*, *TNFAIP3* and *CD247*, have been implicated in the pathogenesis of SSc and may contribute to differences in disease phenotype and severity across racial groups.^73^ Variants in *STAT4* and *IRF5* have been associated with increased susceptibility and pulmonary fibrosis, while *BANK1* polymorphisms are linked to enhanced B-cell activation and diffuse cutaneous disease.^73^

Emerging molecular studies further support ancestry-­associated differences in immune regulation and fibrotic signalling. Epigenetic and transcriptomic profiling of classical monocytes from Black women with SSc identified distinct DNA methylation and gene expression signatures involving metabolic and immune regulatory pathways, further supporting the presence of molecularly distinct disease phenotypes.^74^ These findings suggest that racial disparities in SSc may partially reflect differences in immune regulation and fibrotic signalling, in addition to disparities in healthcare access and socioeconomic factors.

Phenotypic variation across racial groups further supports underlying biologic differences. An earlier onset of Raynaud phenomenon and an increased prevalence of diffuse cutaneous involvement have been seen in Black and Asian patients, where calcinosis and telangiectatic manifestations may differ across populations.^69–71^ Although social determinants of health undoubtedly contribute to delayed diagnosis and worse outcomes, the interaction between immune polarization autoantibody expression and fibrotic progression probably plays a major role in the disproportionate disease burden observed in Black patients with SSc.

## Autoimmune bullous diseases

### Bullous pemphigoid

Bullous pemphigoid (BP) is an autoimmune subepidermal blistering disorder characterized by pruritus, inflammatory plaques and tense bullae resulting from autoantibody-mediated injury to the dermal–epidermal junction.^75–79^ The clinical presentation may differ in patients with skin of colour, in whom erythema can be less apparent and dyspigmentation more prominent.^78^ BP predominately affects older adults, with the incidence increasing significantly after 60 years of age.^80,81^

#### Pathophysiology

BP is driven by autoantibodies targeting the hemidesmosomal proteins BP180 and BP230, leading to complement activation, inflammatory cell recruitment and dermal–epidermal separation.^82–84^ Th2-mediated immune responses play a central role in disease pathogenesis, with elevated IL-4, IL-5 and IL-13, promoting B-cell activation, eosinophilic inflammation and autoantibody production.^85–91^ Additional contributions from Th17 signalling and neutrophil-associated proteases further amplify tissue injury and blister formation.^92^ The efficacy of dupilumab, an IL-4 receptor α antagonist, further supports the importance of Th2 polarization in the pathogenesis of BP.^89^

#### Racial disparities

Racial and ethnic disparities in BP affect disease severity, hospitalization and clinical outcomes. A nationwide paediatric study demonstrated higher hospitalization rates among Black [adjusted odds ratio (aOR) 2.04; *P* = 0.002] and Hispanic (aOR 2.25; *P* < 0.001) patients,^93^ while mortality rates among Black patients were nearly double those found in White patients [0.051 vs. 0.026 per 100 000 population (relative risk 1.95; *P* < 0.001)].^94^ Patients described as ‘non-White’ were also shown to have an increased risk of severe infectious complications (OR 1.20; *P* < 0.001).^95^

Emerging serological and phenotypic data suggest underlying biologic heterogeneity across racial groups. Black patients demonstrate higher Bullous Pemphigoid Disease Area Index scores, particularly within the blistering and dyspigmentation domains.^96^ The combination of elevated BP180 and BP230 autoantibody levels, increased blistering severity and more pronounced dyspigmentation among Black individuals suggests that heightened humoral immune activation may contribute to increased inflammatory injury and greater pigmentation sequelae in patients with skin of color.^96^ In addition, the reduced visibility of erythema and a greater prominence of dyspigmentation in darker skin tones may delay diagnosis and contribute to under-recognition of active inflammation.

Together, these findings suggest that racial disparities in BP probably reflect an interplay between differential immune activation, clinical phenotype variation and inequities in dermatological recognition and care access.

### Pemphigus

Pemphigus is a group of AIBDs characterized by the loss of keratinocyte adhesion and intraepidermal blister formation. Two major forms are pemphigus vulgaris, which commonly involves mucosal erosions and deeper epidermal blistering, and pemphigus foliaceus, which is limited to superficial cutaneous involvement.^97^ Disease severity ranges from localized mucosal disease to widespread erosive involvement associated with significant morbidity.

#### Pathophysiology

Pemphigus is mediated by autoantibodies directed against desmoglein (Dsg) adhesion proteins, which are essential to epidermal integrity.^98,99^ Autoantibody binding induces acantholysis and keratinocyte detachment, resulting in intraepidermal blister formation.^100,101^ Th2-mediated immune responses play a central role in disease pathogenesis, promoting B-cell activation and pathogenic autoantibody production. Increased numbers of CD4^+^ T cells producing IL-4 and IL-10 have been identified in active disease, while murine models demonstrate antigen-specific Th2 induction of Dsg3 autoantibodies.^102–105^

Genetic susceptibility also contributes to significant disease risk. Pemphigus demonstrates strong associations with HLA class II alleles, particularly *HLA-DRB1* and *HLA-DBQ1* variants, which differ across ethnic populations and may influence antigen presentation and autoantibody development.^106^

#### Racial disparities

Significant racial and ethnic disparities exist in the prevalence of pemphigus and the clinical outcomes of patients with the disease. Individuals of Ashkenazi Jewish descent demonstrate the highest disease prevalence, supporting the importance of ancestry-associated genetic susceptibility.^107,108^ Black patients exhibit higher pemphigus-related mortality than White patients (0.030 vs. 0.022 per 100 000 population; *P* < 0.001), while Hispanic, Black, Asian and Native American populations all demonstrate an increased risk of hospital admission for pemphigus-related disease.^94,109^

Emerging evidence suggests that disparities in pemphigus may reflect differences in immune susceptibility and systemic inflammatory burden. Patients described in one study as being ­‘non-White’ demonstrated increased rates of severe infectious complications (OR 1.22; *P* < 0.001),^95^ while Black patients specifically exhibited a higher prevalence of concomitant autoimmune disease (aOR 1.17; *P* = 0.02),^110^ suggesting broader immune dysregulation and autoimmune susceptibility. Together with known HLA-associated risk profiles and Th2-driven autoantibody responses, these findings support the possibility of ancestry-­associated immunological heterogeneity contributing to variation in disease severity and clinical outcomes.

Despite these observations, studies examining racial differences in pemphigus molecular endotypes, cytokine polarization and treatment response remain limited, highlighting an important area for future translational research.

## Secondary lymphoedema

Lymphoedema is a chronic acquired lymphatic disorder characterized by impaired lymphatic drainage, progressive tissue swelling, fibrosis and adipose tissue deposition.^111,112^ In high-­income countries, SL most commonly occurs following cancer treatment, particularly after lymph node dissection and radiation therapy.^112^ Cancer-related SL, especially breast cancer-­related lymphoedema (BCRL), is associated with substantial physical morbidity, recurrent infections, impaired mobility and reduced QoL.^113,114^

### Pathophysiology

SL develops following lymphatic injury caused by surgery, radiation, trauma or infection, resulting in protein-rich fluid accumulation and chronic inflammation.^115,116^ Th2-mediated immune responses play a central role in disease progression, with IL-4 and IL-13 promoting extracellular matrix remodelling, collagen deposition, fibrosis and the inhibition of lymphangiogenesis.^115–118^ Persistent CD4^+^ Th2 activation further amplifies tissue remodelling and lymphatic dysfunction through a self-sustaining inflammatory feedback loop.^115,116,118^

Emerging evidence suggests that SL involves substantial cutaneous and immune alterations beyond mechanical lymphatic obstruction.^119–121^ Increased fibrosis correlates with disease severity and Th2 infiltration in BCRL,^115,116,122^ while Th2 cytokines suppress lymphatic endothelial cell growth and tubule formation, perpetuating swelling and fibrosis.^115,117^ Additional skin changes, including microbial dysbiosis and reduced expression of and epidermal tight junction proteins resembling AD, may further compromise barrier integrity and increase susceptibility to cellulitis and recurrent infection.^121,123,124^

### Racial disparities

Most studies evaluating racial disparities in SL focus on cancer-­related disease, particularly BCRL, where Black and Hispanic patients consistently demonstrate an increased risk of BCRL and disease severity.^125^ In a large Surveillance, Epidemiology, and End Results (SEER)–Medicare cohort of 31 274 patients with breast cancer, Black women had an approximately 50% higher risk of developing BCRL and were nearly twice as likely to develop symptoms within 5 years compared with White women.^125^ Additional studies have similarly demonstrated a significantly higher incidence of BCRL in Black (37.2%) and Hispanic (27.7%) patients compared with White patients (19.8%).^126,127^

Importantly, racial disparities extend beyond disease incidence to include differences in chronicity, fibrosis and QoL. Black patients experience longer symptom duration (11.07 vs. 6.88 years), a greater prevalence of advanced-stage disease (16.7% vs. 8.7%) and worse physical QoL scores than White patients.^128,129^ These findings raise the possibility that heightened inflammatory and fibrotic responses may contribute to more aggressive disease phenotypes, although mechanistic studies specifically evaluating race-associated immune variation in SL remain limited.

At the health-system level, disparities in access to treatment further compound disease burden. Despite the availability of conservative and surgical interventions, Black patients are less likely to have access to adequate therapy (30.8% vs. 55.4%) or surgical management (46.6% vs. 77.2%) than White patients. These disparities may reflect gaps in provider and patient education regarding lymphoedema presentation, as well as delays in diagnosis and referral for appropriate treatment.^128^ Together these findings suggest that racial disparities in SL probably reflect complex interactions between chronic Th2-mediated inflammation, fibrotic remodelling, cutaneous barrier dysfunction and inequities in access to early diagnosis and specialized lymphoedema care.

## Limitations

This study has several limitations. As a narrative review, study selection was not performed using formal systematic review methodology, which may have introduced selection bias. Additionally, the available literature is heterogeneous with respect to study design, patient populations, disease definitions, and the reporting of racial and ethnic categories, limiting direct comparisons across studies. Importantly, data examining racial and ethnic disparities remain limited for several inflammatory skin disorders, particularly with regard to mechanistic, genetic and longitudinal outcomes research, reflecting broader gaps in the existing literature rather than a definitive absence of disparities. Many studies also under-represent minority populations and lack subgroup analyses, restricting the ability to fully characterize immunological and clinical differences across diverse groups. Furthermore, while this review has focused on Th2-mediated immune dysregulation, inflammatory skin diseases are multifactorial and involve complex interactions among genetic, environmental socioeconomic and healthcare access factors that may not be fully captured in the current literature.

## Conclusion

Th2-mediated inflammatory skin diseases are characterized by chronic inflammation, fibrosis and barrier dysfunction, with racial and ethnic disparities influencing disease prevalence, clinical phenotype, immune response and outcomes. Across multiple disorders, Black and Hispanic patients experience disproportionately greater disease burden, more severe infections and increased functional impairment (Table 1). Current molecular and immunological studies indicate that these disparities may reflect not only social and structural inequities, but also differences in immune polarization, cytokine signalling, autoantibody profiles, genetic susceptibility and cutaneous barrier biology.

**Table 1:** Epidemiological, immunological and phenotypic racial disparities across T helper (Th)2-mediated inflammatory skin diseases

Disease	Racial/ethnic group	Epidemiology/burden	Immune/molecular markers	Genotype/serology	Phenotype/clinical outcomes
AD	Black/African American	1.7× higher risk in Black children; higher risk of persistent and severe disease; highest IgE levels reported^31,37,130^	↑ Th2/Th22/Th17 signalling; ↑ IL-22; ↑ IgE^15,16,131,132^	↓ *FLG* loss-of-function variants; ↑ *FLG2* and *CLDN1* mutations^14,21,23–25^	Perifollicular papules, extensor involvement, xerosis and lichenification more commonly described; severity measured using EASI/SCORAD^9–11^
	Hispanic/Latin American	Disproportionate disease burden has been reported in population studies, but group-specific mechanistic data remain limited^1,130^	Race/ethnicity-stratified cytokine data remain limited	Group-specific barrier gene data remain limited	Clinical recognition and treatment disparities may contribute to burden; phenotype-specific data are less well characterized
	Asian	Population-specific disease patterns reported in comparative AD cohorts^30,41^	Increased Th17 polarization compared with classic AD phenotype^41^	*FLG* LoF variants implicated in Asian populations^26–30^	Sharply demarcated, scaly lesions more frequently described^39–41^
	White	Used as the comparator group in US studies	Canonical Th2-mediated AD inflammation is well characterized^13–20^	*FLG* LoF variants strongly associated with AD in European populations^26–28^	Flexural eczema and classic relapsing–remitting pruritus commonly described^9–11^
DLE	Black/African American	Highest Manhattan prevalence among non-Latino Black individuals (23.5); Black-to-White incidence ratio 5.4 in the southeastern USA^48,57^	Limited race-stratified mechanistic data; possible differential immune polarization^54,56^	Group-specific genetic data remain limited; *TYK2*, *IRF5*, *CTLA4* and HLA loci implicated overall^51,52^	Greater odds of scarring alopecia, scalp dyspigmentation and ear dyspigmentation; CLASI used for severity^62^
	Hispanic/Latin American	Manhattan prevalence higher among Latino individuals (8.2) than White and Asian populations^57^	Race/ethnicity-stratified immune profiling remains limited	Group-specific genotype data remain limited	Clinical outcome data are limited; dyspigmentation and delayed recognition may contribute to disease burden in skin of colour
	Asian	Lower Manhattan prevalence reported (0.6)^57^	Group-specific mechanistic data remain limited	Group-specific genotype data remain limited	Phenotype-specific data are limited in available studies
	White	Lower Manhattan prevalence reported (1.8) compared with non-Latino Black and Latino groups^57^	Comparator group in available epidemiological studies	*TYK2*, *IRF5*, *CTLA4* and HLA susceptibility loci reported in DLE overall^51,52^	Discrete inflammatory plaques may progress to dyspigmentation, atrophy, scarring and telangiectasia^46^
SSc	Black/African American	Younger age at onset/diagnosis; more frequent dSSc; more severe pulmonary fibrosis and higher mRSS^69–72^	Distinct immune endotypes; monocyte DNA methylation and gene expression signatures involving metabolic and immune regulatory pathways^74^	↑ Anti-topoisomerase I (26% vs. 17%), anti-U1 RNP (18% vs. 5%) and anti-U3 RNP (19% vs. 3%) compared with White patients^74^	More aggressive fibrotic phenotype, internal organ involvement, skeletal muscle involvement and functional impairment^70–72^
	Asian	Earlier RP and increased diffuse cutaneous involvement have been observed^69–71^	Immune and fibrotic pathway data by subgroup remain limited	HLA-linked autoantibody variation may differ across ethnic groups^73^	Calcinosis and telangiectatic manifestations may vary across populations^69–71^
	White	Often comparator group; lower frequency of diffuse disease and selected autoantibodies compared with Black patients in cited cohorts^70–72^	Comparator for molecular and serological studies	Lower anti-topoisomerase I, anti-U1 RNP and anti-U3 RNP frequencies compared with Black patients in cited cohort^74^	SSc manifests with skin thickening, RP, fibrosis and internal organ involvement; mRSS used for severity^63,64^
	Multiethnic/overall	Phenotype and outcomes vary across cohorts	Th2 cytokines IL-4 and IL-13, IL-6, TGF-β, endothelin-1, Th17 and Tfh pathways implicated^65–68^	*STAT4*, *IRF5*, *BANK1*, *TNFAIP3* and *CD247* implicated in susceptibility and fibrotic/immune signalling^73^	Disease ranges from limited to diffuse cutaneous involvement with variable visceral disease^63–65^
BP	Black/African American	Mortality nearly double that of White patients; higher BPDAI blistering and dyspigmentation domains reported^94,96^	Heightened Th2-mediated humoral activation suggested^85–88,96^	Elevated BP180 and BP230 autoantibody levels reported^82,83,96^	Greater blistering severity and dyspigmentation; erythema may be less apparent in darker skin tones^78,96^
	Hispanic/Latin American	Higher odds (2.25) of paediatric hospitalization^93^	Group-specific mechanistic data remain limited	Group-specific BP180/BP230 data remain limited	Clinical outcome data remain limited; hospitalization burden increased in paediatric analysis^93^
	White	Comparator mortality rate 0.026 per 100 000 in cited mortality study^94^	Comparator group in racial disparity studies	BP180/BP230 autoantibodies remain central diagnostic markers^82–84^	Pruritic patches and tense bullae; BPDAI used for severity^76,79,96^
Pemphigus	Ashkenazi Jewish ancestry	Highest disease prevalence reported, supporting ancestry-associated susceptibility^107,108^	Th2-driven autoantibody responses implicated^102,133^	Strong HLA class II associations, especially *HLA-DRB1* and *HLA-DQB1* variants^106^	PV involves mucosal erosions; PF involves superficial cutaneous disease; PDAI used for severity^97^
	Black/African American	Higher pemphigus-related mortality than White patients; higher concomitant autoimmune disease reported^94,110^	Broader immune dysregulation and autoimmune susceptibility suggested^110^	Group-specific HLA data remain limited in available studies	Higher mortality and comorbidity burden; hospitalization risk elevated among Black patients^94,109,110^
	Hispanic/Latin American, Asian, and Native American	Higher risk of pemphigus-related hospital admission reported^109^	Group-specific cytokine/endotype data remain limited	*HLA-DRB1*/*HLA-DQB1* susceptibility varies across ethnic populations^106^	Clinical outcomes include erosive cutaneous and mucosal disease; hospitalization burden increased^97,109^
LE	Black/African American	Approximately 50% higher BCRL risk in SEER–Medicare cohort; 37.2% incidence vs. 19.8% in White patients in additional studies^125–127^	Race-specific immune profiling limited; heightened inflammatory/fibrotic responses may contribute to aggressive phenotypes^115–118,128^	No consistent race-specific genotype/serology markers reported	Longer symptom duration, advanced ISL stage, worse physical QoL and lower receipt of maximal therapy or surgery^128,129^
	Hispanic/Latin American	Higher BCRL incidence reported (27.7%) vs. White patients (19.8%)^126,127^	Race-specific immune profiling remains limited	No consistent race-specific genotype/serology markers reported	Increased BCRL burden; access to early diagnosis and specialized care may influence outcomes^125–127^
	White	Comparator BCRL incidence 19.8%; shorter symptom duration and lower advanced-stage prevalence than Black patients^126–129^	Th2 fibrosis and lymphatic dysfunction pathways apply to disease overall^115–118^	No consistent race-specific genotype/serology markers reported	Progressive swelling, fibrosis, recurrent infections, impaired mobility and reduced QoL^111–114^

AD, atopic dermatitis; BCRL, breast cancer-related lymphoedema; BP, bullous pemphigoid; BPDAI, Bullous Pemphigoid Disease Area Index; CLASI, Cutaneous Lupus Erythematosus Disease Area and Severity Index; DLE, discoid lupus erythematosus; dSSc, diffuse cutaneous systemic sclerosis; EASI, Eczema Area and Severity Index; HLA, human leucocyte antigen; IL, interleukin; ISL, International Society of Lymphology; LE, secondary lymphoedema; LoF, loss of function; mRSS, modified Rodnan skin score; PDAI, Pemphigus Disease Area Index; PF, pemphigus foliaceus; PV, pemphigus vulgaris; QoL, quality of life; RNP, ribonucleic protein; RP, Raynaud phenomenon; SCORAD, SCORing Atopic Dermatitis; SEER, Surveillance, Epidemiology, and End Results; SSc, systemic sclerosis; Tfh, T follicular helper; TGF-β, transforming growth factor-β.

Although socioeconomic factors and inequitable access to dermatological care remain major contributors to delayed diagnosis and worse outcomes, growing molecular and immunological data support the presence of ancestry-associated disease heterogeneity across several Th2-mediated disorders. However, substantial gaps persist in understanding racial differences in immune endotypes, epidermal gene expression, microbiome composition and response to targeted biologic therapies. Under-representation of diverse populations in translational studies and clinical trials further limits the ability to characterize disease mechanisms and optimize treatment strategies across patient populations.

Addressing these disparities will require molecular, genetic and population-level research alongside interventions aimed at improving healthcare access and representation in dermatological research. Greater inclusion of historically under-represented populations will be essential to advancing precision dermatology and reducing inequities in the diagnosis, treatments and outcomes of Th2-mediated inflammatory skin diseases.

## Data Availability

The data underlying this article are available in the article.
